# Resistance Exercise Reduces Kynurenine Pathway Metabolites in Breast Cancer Patients Undergoing Radiotherapy

**DOI:** 10.3389/fonc.2019.00962

**Published:** 2019-09-25

**Authors:** Philipp Zimmer, Martina E. Schmidt, Mirja Tamara Prentzell, Bianca Berdel, Joachim Wiskemann, Karl Heinz Kellner, Jürgen Debus, Cornelia Ulrich, Christiane A. Opitz, Karen Steindorf

**Affiliations:** ^1^Division of Physical Activity, Prevention and Cancer, German Cancer Research Center (DKFZ) and National Center for Tumor Diseases (NCT), Heidelberg, Germany; ^2^Department for Molecular and Cellular Sports Medicine, Institute for Cardiovascular Research and Sports Medicine, German Sport University Cologne, Cologne, Germany; ^3^DKTK Brain Cancer Metabolism Group, German Cancer Research Center (DKFZ), Heidelberg, Germany; ^4^Department of Medical Oncology, National Center for Tumor Diseases (NCT), University Hospital Heidelberg, Heidelberg, Germany; ^5^Neuroimmun GmbH, Karlsruhe, Germany; ^6^Department of Radiation Oncology, National Center for Radiation Oncology (NCRO), Heidelberg Institute for Radiation Oncology (HIRO), University Hospital Heidelberg, Heidelberg, Germany; ^7^Department of Population Health Sciences, Huntsman Cancer Institute, University of Utah, Salt Lake City, UT, United States; ^8^Department of Neurology and National Center for Tumor Diseases (NCT), University Hospital Heidelberg, Heidelberg, Germany

**Keywords:** exercise, physical activity, cancer, tryptophan, kynurenine, kynurenic acid, quinolinic acid

## Abstract

**Purpose:** Evidence from preclinical studies and trials in healthy volunteers suggests that exercise may modulate the levels of tryptophan (TRP) metabolites along the kynurenine (KYN) pathway. As KYN and downstream KYN metabolites are known to promote cancer progression by inhibiting anti-tumor immune responses and by promoting the motility of cancer cells, we investigated if resistance exercise can also control the levels of KYN pathway metabolites in breast cancer patients undergoing radiotherapy (NCT01468766).

**Patients and Methods:** Chemotherapy-naïve breast cancer patients (*n* = 96) were either randomized to an exercise/intervention group (IG) or a control group (CG). The IG participated in a 12-week supervised progressive resistance exercise program twice a week, whereas the CG received a supervised relaxation program. Serum levels of TRP and KYN as well as urine levels of kynurenic acid (KYNA) and neurotoxic quinolinic acid (QUINA) were assessed before (t0), after radiotherapy, and mid-term of the exercise intervention (t1) and after the exercise intervention (t2). Additionally, 24 healthy women (HIG) participated in the exercise program to investigate potential differences in its effects on KYN metabolites in comparison to the breast cancer patients.

**Results:** At baseline (t0) the breast cancer patients showed a significantly elevated serum KYN/TRP ratio and urine QUINA/KYNA ratio, as well as increased urine QUINA levels in comparison to the healthy women. In response to exercise the healthy women and the breast cancer patients differed significantly in the levels of urine QUINA and the QUINA/KYNA ratio. Most importantly, serum KYN levels and the KYN/TRP ratio were significantly reduced in exercising patients (IG) compared to non-exercising patients (CG) both at t1 and t2.

**Conclusion:** Resistance exercise may represent a potent non-pharmacological avenue to counteract an activation of the KYN pathway in breast cancer patients undergoing radiotherapy.

## Introduction

Increased levels of kynurenine (KYN), a catabolite of the amino acid tryptophan (TRP), are associated with progression and poor clinical outcome in numerous cancer types (1). The degradation of TRP to KYN is catalyzed by the isoenzymes tryptophan 2,3-dioxygenase (TDO) and indoleamine 2,3-dioxygenase 1 and 2 (IDO1, IDO2). Cancers often express high levels of IDO1 and/or TDO and TRP metabolism represents a critical metabolic pathway controlling tumor immune evasion as well as tumor cell malignant properties (2–7). Inhibitors of IDO1 and TDO as well as downstream modulators of the KYN pathway and its targets are therefore being developed for cancer immunotherapy and are advancing into clinical trials. KYN as well as its downstream metabolites kynurenic acid (KYNA) and quinolinic acid (QUINA) mediate various inhibitory effects on the immune system including suppression of T-cell proliferation, the differentiation of regulatory T-cells, suppression of Th17 cells, promotion of tolerogenic dendritic cells (DC), and an impaired function of effector cells such as natural killer (NK) cells and cytotoxic T-cells (8–14).

Regular physical exercise is associated with decreased cancer risk and progression (15). It has been speculated that these positive effects of exercise are partially mediated by influencing the body's (anti-) inflammatory homeostasis. In contrast to a single bout of exercise, which induces short-term systemic inflammation, long-term regular physical exercise is known to reduce systemic inflammation (16). As IDO1 and TDO are induced by pro-inflammatory stimuli (17, 18), distinct exercise modalities may hence differentially influence their activity and downstream effects.

Apart from cancer, a dysregulation of the KYN pathway has also been described for neuropsychiatric and neurodegenerative disorders, such as depression, Alzheimer's disease, and multiple sclerosis (19, 20). These effects of the KYN pathway in the central nervous system (CNS) may also be relevant for oncology, as many cancer patients suffer from depressive symptoms, fatigue, and cognitive impairments (21, 22). Elevated blood levels of KYN have been shown to induce depressive symptoms in mice (23) and have been measured in depressive patients (24). KYN generated by tumors or the tumor microenvironment is able to cross the blood brain barrier (25) and can further be metabolized to QUINA or KYNA in the CNS. QUINA stimulates excitatory N-methyl-D-aspartate (NMDA) receptors and can induce neuronal excitotoxicity, while KYNA inhibits NMDA-, kainate-, and α-amino-3-hydroxy-5-methyl-4-isoxazolepropionic acid (AMPA) receptors, in addition to negatively modulating α7 nicotinic receptors (26, 27). Due to their opposing neuroactive properties, alterations in the balance between QUINA and KYNA are considered to play an important role in neurodegenerative and neuropsychiatric diseases (26–28).

Regular physical exercise has been described as a promising (supportive) non-pharmacological treatment to counteract the development and progression of neurological disorders (29–32). In regard to cancer, accumulating evidence suggests that exercise reduces fatigue, depressive symptoms, and cancer related cognitive impairment (15, 33). Apart from the anti-inflammatory effects of physical exercise, Agudelo et al. have shown that physical exercise induces the expression of specific isoforms of the enzyme KYN amino transferase (KAT) in skeletal muscle of mice, leading to an enhanced peripheral conversion of KYN to KYNA (23). As KYNA is poorly able to cross the blood brain barrier (25), the conversion of KYN to KYNA in the periphery was thought to decrease CNS KYN levels and thus reduce neuroinflammation as well as depressive symptoms.

In light of the anti-inflammatory properties of physical exercise and the important role of skeletal muscle as a modifier of the KYN pathway, we investigated the effect of a supervised 12-week resistance exercise program on TRP and downstream TRP metabolites in breast cancer patients undergoing radiotherapy.

## Materials and Methods

The study was approved by the Ethics Committee of the University of Heidelberg (NCT01468766) and complied with the principles of the Declaration of Helsinki. Written informed consent was obtained from all participants.

### General Procedure

Interested women underwent a baseline assessment, including a check for contraindications for resistance training, e.g., acute infectious disease, severe cardiac disease, severe respiratory insufficiency, as well as an ECG at rest followed by an endurance performance test on a cycle ergometer in combination with an exercise ECG. Women were included only if there were no concerns regarding progressive resistance training. Chemotherapy-naïve breast cancer patients (*n* = 96) were randomized into either an exercise/intervention group (IG) or a control group (CG). Allocation was done by a biometrician who was not involved in the recruitment procedure, based on predetermined lists with random block size, stratified by age and baseline physical fatigue level [fatigue was the primary outcome of this trial (34)]. Study personnel did not have access to the randomization lists. The IG participated in a 12-week supervised progressive resistance exercise program twice a week starting in parallel to adjuvant radiotherapy, whereas the CG received a supervised relaxation program (34). Resting blood and urine samples were taken prior to (t0), after completion of the 6-week radiation therapy i.e., in the middle of the exercise intervention (t1), and after completing the 12-week exercise program (t2). In addition, 24 healthy women (HIG) were recruited to participate in the same exercise program as the IG.

### In- and Exclusion Criteria

The in- and exclusion criteria were defined as follows: patients were 18 years or older, diagnosed with breast cancer (stage 0–3), had not received adjuvant or neo-adjuvant chemotherapy, did not have physical impairment preventing participation in the exercise program (e.g., acute infectious disease, severe cardiac disease, severe respiratory insufficiency), and no other concomitant malignant diseases. Healthy subjects of comparable age were included that never had cancer, any other chronic internal disease or orthopedic impairment preventing participation in the exercise program.

### Intervention

A detailed description of the exercise intervention was already published (35, 36). In brief, the 12-week whole body resistance exercise program was conducted twice weekly for 1 h and consisted of eight different, machine-based exercises (leg extension; leg curl; leg press; shoulder internal and external rotation; seated row; latissimus pull down; shoulder flexion and extension and butterfly and butterfly reverse). Patients performed three sets with a maximum of twelve repetitions (60–80% of the one repetition maximum) and 1 min resting periods in between sets. Training was progressive in terms of weight increase to the next machine weight level (at least by 5%) after successfully completing three sets of an exercise with 12 repetitions in three consecutive sessions. The control group participated in a progressive muscle relaxation training according to Jacobsen, which also took place twice weekly for 1 h.

### Outcome Measures

Serum TRP and KYN levels as well as urine KYNA and QUINA levels were analyzed for all time points by Enzyme-linked Immunosorbent Assay (ELISA) according to the manufacturer's instructions using the following ELISA kits: #K7726, #K7735, and #K7736 (Neuroimmun GmbH, Germany).

The performance characteristics of the ELISAs are as follows:

**#K7726**

Precision and Reproducibility for L-kynurenine

**Table:** 

Intra-Assay (*n* = 14)		
Sample	L-kynurenine [μmol/l]	CV [%]
1	0.82	7.6
2	2.86	6.2
Inter-Assay (*n* = 8)		
Sample	L-kynurenine [μmol/l]	CV [%]
1	0.80	
2	2.80	6.2

Spiking Recovery

Three samples were spiked with different L-kynurenine concentrations and measured in this assay (*n* = 2). The mean recovery rate was 102.5%.

Dilution Recovery

Two spiked samples were diluted and analyzed. The mean recovery rate was 100.3% (*n* = 2).

Analytical Sensitivity

Limit of detection, LoD 0.12 μmol/l

Precision and Reproducibility for L-tryptophan

**Table:** 

Intra-assay (*n* = 14)		
Sample	L-tryptophan [μmol/l]	CV [%]
1	51.4	4.3
2	105.7	6.9
Inter-assay (*n* = 7)		
Sample	L-tryptophan [μmol/l]	CV [%]
1	63.7	8.4
2	60.6	9.1

Spiking Recovery

Three serum samples were spiked with different L-tryptophan concentrations and measured in this assay (*n* = 2). The mean recovery rate for all concentrations was 97.2%.

Dilution Recovery

Two serum samples were diluted and measured in this assay. The mean recovery was 96.8% (*n* = 2).

Analytical Sensitivity

Limit of detection, LoD 8.0 μmol/l

**#K7735**

Precision and Reproducibility for Kynurenic Acid

**Table:** 

Intra-assay (*n* = 24)		
Sample	Kynurenic acid [μmol/l]	CV [%]
1	28.8	5.7
2	26.9	8.0
Inter-assay (*n* = 7)		
Sample	Kynurenic acid [μmol/l]	CV [%]
1	15.9	11.4
2	33.0	9.8

Spiking Recovery

Three urine samples were spiked with different kynurenic acid concentrations and measured in this assay. The mean recovery rate was 95.8% (*n* = 2).

Dilution Recovery

Three urine samples were diluted with assay buffer and measured in this assay. The mean recovery rate was 106.8% (*n* = 2).

Analytical Sensitivity

The zero-standard was measured 48 times. The detection limit was set as B_0_−2 SD and estimated to be 0.3 μmol/l.

**K7736**

Precision and Reproducibility for Quinolinic Acid

**Table:** 

Intra-assay (*n* = 8)		
Sample	Quinolinic acid [μmol/l]	CV [%]
1	32.03	7.1
2	66.99	5.9
Inter-assay (*n* = 10)		
Sample	Quinolinic acid [μmol/l]	CV [%]
1	74.05	4.3
2	49.34	5.8

Spiking Recovery

Three urine samples were spiked with different quinolinic acid concentrations and measured in this assay. The mean recovery rate was 101.0% (*n* = 2).

Dilution Recovery

Two urine samples were diluted with assay buffer and measured in this assay. The mean recovery rate was 101.0% (*n* = 2).

Analytical Sensitivity

The zero-standard was measured 50 times. The detection limit was set as B_0_−2 SD and estimated to be 2.8 μmol/l.

Analysis of ELISA values were performed with the Magellan software V 7.2 (Tecan Austria GmbH). Urine KYNA and QUINA were normalized to Creatinine. The KYN/TRP ratio was calculated to indirectly assess TRP degradation. The KYNA/KYN ratio was used as indicator for KAT activity. The QUINA/KYNA ratio was calculated as parameter to indirectly assess neurotoxicity.

### Statistics

All statistical analysis were conducted using SPSS 25 (IBM). First, *t*-Tests were used to compare baseline values of all outcomes between healthy women and breast cancer patients. Second, a baseline-adjusted 2 (groups: IG, HIG) × 2 (t0, t2) ANCOVA was used to investigate potential differences of the exercise program on the KYN pathway between breast cancer patients (IG) and healthy subjects (HIG). Third, a baseline- and age-adjusted 2 (groups: IG, CG) × 3 (time points: t0, t1, t2) ANCOVA was conducted to determine differences for changes in each outcome variable between IG and CG. Significant time effects and group x time interactions were further analyzed by Bonferroni corrected simple effects analysis. Prior to ANCOVA a z-transformation was conducted to identify outliers (> +/– 3 SD). For all analysis alpha was set at 0.05.

## Results

To investigate the effect of a supervised 12-week resistance exercise program on TRP metabolism, we measured TRP and KYN in the serum and KYNA and QUINA in the urine of healthy women as well as breast cancer patients undergoing radiotherapy in the BEST study (34). The baseline characteristics of the breast cancer patients (*n* = 96) who were randomized to the intervention group (IG) and control group (CG) as well those of the healthy intervention group (HIG, *n* = 24) are shown in Table 1. Overall adherence was similar in IG and CG with a median of 20 out of 24 scheduled visits.

**Table 1:** Baseline characteristics.

		**Intervention group**		**Control group**		**Healthy intervention group**	
**TOTAL** ***n***		52		44		24	
Age, mean (SD)		57.3	(8.8)	56.7	(9.0)	53.1	(10.0)
BMI, mean (SD)		26.5	(4.6)	27.9	(5.5)	24.1	(4.3)
Smoker	Non	40	(76.9)	40	(90.9)	20	(80)
	Ex	4	(7.7)	1	(2.3)	–	–
	Current	8	(15.4)	3	(6.8)	4	(20)
Days since surgery mean (SD)		44.8	(14.0)	44.4	(11.7)	–	
Breast surgery *n* (%)	Ablation	2	(3.8)	2	(4.5)	–	
	Lumpectomy	50	(96.2)	42	(95.5)	–	
Stage, *n* (%)	0	7	(13.5)	5	(11.4)	–	
	1	30	(57.7)	32	(72.7)	–	
	2a	12	(23.1)	6	(13.6)	–	
	2b	2	(3.8)	1	(2.3)	–	
	3a	1	(1.9)	–	–	–	
Hormone therapy *n* (%)	No	22	(42.3)	22	(50.0)	–	
	Yes	30	(57.7)	22	(50.0)	–	
Sports before diagnosis *n* (%)	None	24	(46.2)	16	(36.4)	8	(32)
	>0–9 MET*h/wk	14	(26.9)	12	(27.3)	13	(52)
	>9–21 MET*h/wk	9	(17.3)	7	(15.9)	4	(16)
	>21 MET*h/wk	5	(9.6)	9	(20.5)	0	0

*Baseline characteristics of patients of the intervention and the control group (IG and CG) as well as those of the healthy intervention group (HIG). Data are presented as means and standard deviation or percentages where appropriate. Metabolic Equivalent of Task (MET) hours (h)/week (wk)*.

### Baseline Comparison Between Patients With Breast Cancer and Healthy Subjects

**Figure 1:**
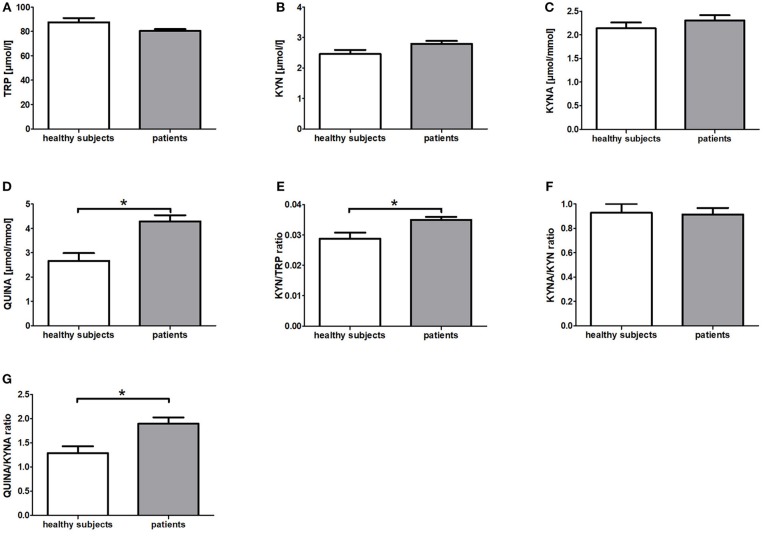
Baseline differences of KYN metabolites and their corresponding ratios between patients with breast cancer and healthy subjects. Data are presented as means +/− standard error of the mean. Significant baseline differences of TRP metabolites and their corresponding ratios **(A–G)** between patients with breast cancer and healthy subjects are marked by *.

In comparison to the healthy subjects (HIG) the breast cancer patients (IG + CG) showed significantly elevated serum KYN/TRP ratios (*p* = 0.006) as well as urine QUINA levels (*p* = 0.003) and QUINA/KYNA ratios (*p* = 0.003) at baseline (t0) (Figure 1). Moreover, a tendency toward reduced serum TRP levels (*p* = 0.051) and increased serum KYN levels (*p* = 0.084) was observed in the patients (Figure 1). No differences were observed for KYNA levels (*p* = 0.308) and the KYNA/KYN ratio (*p* = 0.901) (Figure 1).

### Comparison of the Responses to the Resistance Training Between Breast Cancer Patients and Healthy Subjects

**Figure 2:**
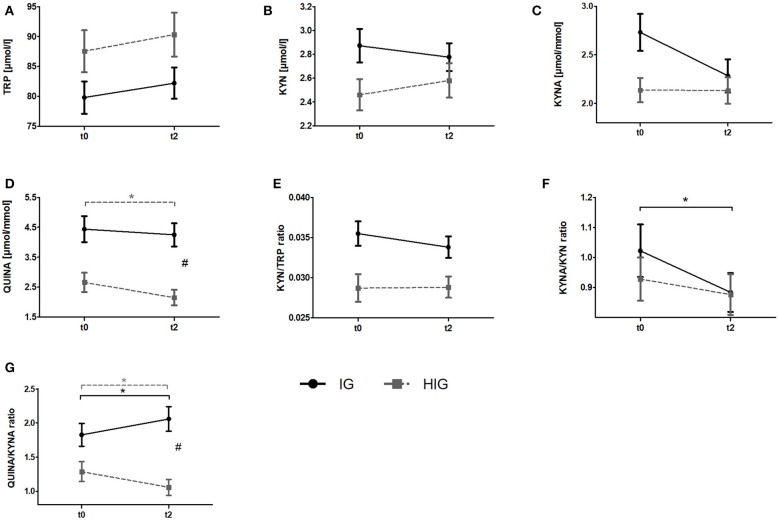
Comparison of responses to the resistance exercise program between patients with breast cancer and healthy subjects. Raw data are presented as means +/− standard error of the mean. IG, intervention group (black); HIG, healthy intervention group (gray); t0, before radiation and exercise program; t2, end of intervention/6 weeks after completing radiation. **(A)** Tryptophan (TRP) **(B)** Kynurenine (KYN), **(C)** Kynurenic acid, **(D)** QUINA, **(E)** KYN/TRP ratio, **(F)** KYNA/KYN ratio, **(G)** QUINA/KYNA ratio. *Post-hoc* results for 2 × 2 ANCOVA: *significant within-group differences. #significant between-group differences.

To investigate whether resistance exercise differentially affects healthy individuals and breast cancer patients, we compared serum TRP and KYN levels as well as urine QUINA and KYNA levels between the IG and the HIG at t0 and t2. Raw data and time courses of all outcome measures as well as *post-hoc* results of 2 (IG, CG) × 2 (t0, t2) ANCOVA are shown in Figure 2. ANCOVA results are listed in Table 2.

**Table 2:** ANCOVA results for all outcome measures.

**Outcome**	**Group**	***n***	**ANCOVA time**	**ANCOVA group × time**
TRP [μmol/l]	IG	43	*F =* 3.819, df = 1.788 ***p*** **=** **0.024**	*F =* 0.049, df = 0.193 *p* = 0.801
	CG	42		
	HIG	24	*F =* 15.031, df = 1 ***p*** **<** **0.001**	*F =* 0.749, df = 1 *p* = 0.390
KYN [μmol/l]	IG	44	*F =* 0.788, df = 1.806 *p* = 0.445	*F =* 5.624, df = 1.806 ***p*** **=** **0.006**
	CG	41		
	HIG	24	*F =* 21.643, df = 1 ***p*** **<** **0.001**	*F =* 23.416, df = 1 *p* = 0.661
KYNA [μmol/mmol]	IG	35	*F =* 2.844, df = 2 *p* = 0.062	*F =* 1.507, df = 2 *p* = 0.225
	CG	39		
	HIG	24	*F =* 48.235, df = 1 ***p*** **<** **0.001**	*F =* 0.086, df = 1 *p* = 0.770
QUINA [μmol/mmol]	IG	36	*F =* 0.991, df = 2 *p* = 0.374	*F =* 0.080, df = 2 *p* = 0.923
	CG	34		
	HIG	23	*F =* 19.901, df = 1 ***p*** **<** **0.001**	*F =* 7.536, df = 1 ***p*** **=** **0.008**
KYN/TRP	IG	41	*F =* 0.952, df = 2 *p* = 0.388	*F =* 6.120, df = 2 ***p*** **=** **0.003**
	CG	40		
	HIG	24	*F =* 31.194, df = 1 ***p*** **<** **0.001**	*F =* 1.185, df = 1 *p* = 0.280
KYNA/KYN	IG	31	*F =* 4.396, df = 2 ***p*** **=** **0.014**	*F =* 3.009, df = 2 *p* = 0.053
	CG	37		
	HIG	24	*F =* 0.47.959, df = 1 ***p*** **<** **0.001**	*F =* 0.205, df = 1 *p* = 0.653
QUINA/KYNA	IG	33	*F =* 1.132, df = 1.270 *p* = 0.305	*F =* 1.296, df = 1.270 *p* = 0.268
	CG	32		
	HIG	23	*F =* 12.506, df = 1 ***p*** **=** **0.001**	*F =* 11.579, df = 1 ***p*** **=** **0.001**

*IG, breast cancer intervention group; CG, breast cancer control group; HIG, healthy intervention group; n, number of individuals. Results of 2 (IG, CG) × 3 (t0, t1, t2) and 2 (IG, HIG) × 2 (t0, t2) ANCOVAS for Tryptophan (TRP), Kynurenine (KYN), Kynurenic acid (KYNA), Quinolinic acid (QUINA), and corresponding ratios. Baseline and age adjusted ANCOVA results are presented as F-values, degrees of freedom (df) and p-values*.

ANCOVA indicated significant time effects for all outcome measures. However, *post-hoc* analysis indicated only significant decreases for QUINA levels (*p* = 0.004) and the QUINA/KYNA (*p* = 0.040) ratio in healthy exercising individuals. In contrast QUINA/KYNA ratio increased in the patient group (*p* = 0.007). For QUINA levels and QUINA/KYNA ratio ANCOVA additionally showed significant group x time interactions. *Post-hoc* tests indicated significantly elevated QUINA levels (*p* = 0.008) and QUINA/KYNA ratio (*p* = 0.001) at t2 in the patient population.

### Impact of Resistance Exercise on the KYN Pathway in Patients With Breast Cancer

**Figure 3:**
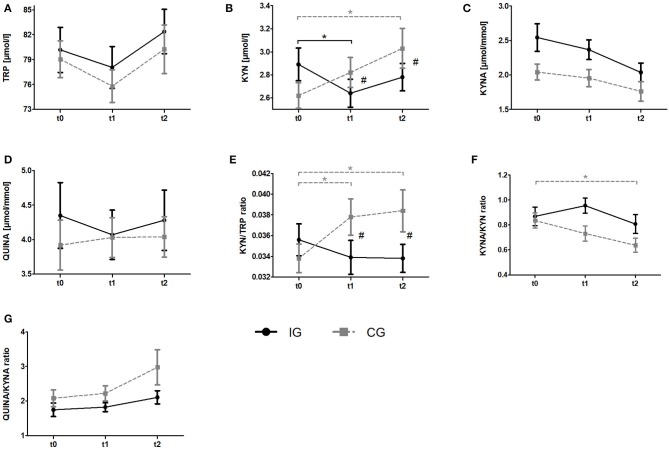
Impact of resistance exercise on the KYN pathway in breast cancer patients undergoing radiotherapy. Raw data are presented as means +/– standard error of the mean. IG, intervention group (black); CG, control group (gray); t0, before radiation and exercise program; t1, mid-intervention/after radiotherapy (t0 + 6 weeks); t2, end of intervention/6 weeks after completing radiation. **(A)** Tryptophan (TRP) **(B)** Kynurenine (KYN), **(C)** Kynurenic acid, **(D)** QUINA, **(E)** KYN/TRP ratio, **(F)** KYNA/KYN ratio, **(G)** QUINA/KYNA ratio. *Post-hoc* results for 2 × 3 ANCOCA: *significant within-group differences. #significant between-group differences.

To analyze the effects of a resistance exercise program on the KYN pathway in breast cancer patients, we compared serum TRP and KYN levels as well as urine QUINA and KYNA levels between the IG and the CG at t0, t1, and t2. Raw data and time courses of all outcome measures as well as *post-hoc* results of 2 (IG, CG) × 3 (t0, t1, t2) ANCOVA are shown in Figure 3. ANCOVA results are listed in Table 2.

Regarding Trp, ANCOVA revealed a significant time effect. However, *post-hoc* analysis revealed no significant within-group changes over time. KYN levels indicated a significant group x time interaction. *Post-hoc* analysis showed a decrease of KYN levels from baseline to t1 (*p* = 0.036) for the IG whereas the CG showed a constant increase over time which became significant comparing t0 and t2 (*p* = 0.002). Groups significantly differ at t1 (*p* = 0.003) and t2 (*p* = 0.003). For KYNA and QUINA neither time effects nor group × time interactions were found. In view of Kyn/Trp ratio, ANCOVA revealed a significant group × time interaction. Subsequent *post-hoc* tests indicated a significant increase of KYN/TRP ratio from baseline to t1 (*p* = 0.009) and to t2 (*p* = 0.006) in the CG whereas no alterations were detected in the IG. Again, groups significantly differed at t1 (*p* = 0.003) and t2 (*p* = 0.003). For KYNA/KYN ratio ANCOVA revealed a significant time effect. Subsequent *post-hoc* analysis revealed a significant decrease of this ratio from t0 to t2 (*p* = 0.002) in the CG whereas no changes were observed in the IG. Although a tendency for an interaction was observed in the ANCOVA it does not reach statistical significance (*p* = 0.053). In view of the QUINA/KYNA ratio neither time effects nor interactions were detected.

## Discussion

To our knowledge, this is the first clinical trial investigating the influence of resistance exercise on the KYN pathway. Specifically, we investigated the potential exercise-induced modulation of KYN pathway metabolites in the serum and urine of healthy women and breast cancer patients undergoing radiotherapy. At baseline (t0) the KYN/TRP ratio was elevated in breast cancer patients (IG + CG) compared to healthy women (Figure 1). This finding is in line with a previous study reporting increased TRP metabolism in comparison to healthy controls in breast cancer patients as well as in patients with other cancer entities such as non-small cell lung cancer and adult T cell leukemia/lymphoma (37, 38). Moreover, QUINA levels were significantly elevated in the urine of breast cancer patients in comparison to healthy women (Figure 1), which likely is a direct consequence of the enhanced TRP degradation in these patients. Elevated QUINA levels and QUINA/KYNA ratios in patients may also be the consequence of an inflammation driven induction of kynurenine 3-monooxygenase (KMO) (39). Comparison of the effects of resistance exercise between breast cancer patients (IG) and healthy women (HIG) revealed that while urine QUINA levels and the QUINA/KYNA ratio were reduced in the healthy subjects (HIG), breast cancer patients (IG) showed reduced KYNA/KYN ratios and elevated QUINA/KYNA ratios after completion of the 12-week exercise training (Figure 2). It is not clear why these two populations showed opposite effects of exercise on these downstream KYN pathway metabolites. However, it is important to acknowledge that the two populations not only differed in their disease state but that the breast cancer patients received 6-weeks of radiotherapy during the exercise intervention, which may have contributed to the observed effects on the KYN pathway metabolites.

Interestingly, breast cancer patients who did not exercise (CG) showed a significant and sustainable increase in serum KYN levels and the KYN/TRP ratio after completing radiotherapy (Figure 3). This increase is likely mediated by the radiotherapy, causing tissue damage and subsequent inflammation, which is known to potently induce IDO1 (40). Of note, resistance exercise (IG) counteracted this increase in circulating KYN levels (Figure 3). Previous results from this study and many others suggest that exercise reduces resting levels of inflammatory markers, such as IL-6 (41) and CRP (42). The lower levels of inflammatory cytokines may result in reduced IDO1 expression thus explaining the effect of exercise on the KYN/TRP ratio. However, it remains speculative whether the detected effect of resistance exercise on the KYN/TRP ratio is exclusively related to less expression of IDO1. Exercise is also known to suppress (43) the levels of cortisol (e g., in people with depressive disorders), a well-described inducer of TDO, suggesting that exercise could also downregulate this TRP degrading enzyme (44, 45).

The exercise-induced decrease in resting KYN levels seems to be in line with some of its immune-modulatory properties. Thus far, it was demonstrated that NK-cell cytotoxicity is increased in patients with breast cancer after participating in an endurance exercise program (46). As mentioned in the introduction, NK-cell cytotoxicity is known to be suppressed by increased KYN levels. Additionally, enhanced physical capacity (reached by regular training) is associated with elevated numbers and proportions of regulatory T-cells in the peripheral blood of healthy subjects (47). Regulatory T-cells are known to be induced by elevated KYN levels (48). These findings can be reconciled with our results, as each single bout of physical exercise induces short-term inflammatory processes, including subsequently elevated KYN levels (49, 50). Therefore, a long-term increase in regulatory T-cells may represent the body's response to repeated (short-term exercise-induced) inflammatory stress leading to transiently elevated KYN levels. In contrast, exercise is not expected to increase regulatory T-cells in the tumor microenvironment (51).

Despite reducing serum KYN levels, resistance exercise did not alter the concentrations or ratios of KYNA and QUINA in the urine of the breast cancer patients. Currently it is unclear how the urine levels of these TRP metabolites are associated with the corresponding blood and CNS levels. Agudelo et al. reported an increased conversion of KYN to KYNA after physical exercise in rodents (23). The authors described that muscle KAT4 expression was stimulated by PGC-1α activation in response to endurance exercise. In line with our findings in urine, Millischer et al. reported no changes of the plasma KYNA/KYN ratio in depressive patients in response to varying 12-week home-based exercise programs (yoga like, moderate endurance exercise, intensive mixed endurance, and strength training) (52). The authors hypothesized that relevant alterations of KAT expression are rather expected acutely after exercise, since PGC-1α returns to baseline levels within 24 h after cessation of physical activity. Indeed, this hypothesis is partially confirmed by the results of Schlittler et al. who found increased KAT levels in skeletal muscle in response to a single bout of endurance exercise (53).

Cancer patients frequently suffer from disease- and treatment-related adverse effects, such as cognitive impairment and fatigue which have a certain behavioral and biological (e.g., increased inflammation) overlap with major depression (15). We and others have previously shown that physical exercise during and after medical treatment is able to reduce fatigue (34, 54, 55). Further research is necessary to establish if exercise-induced changes in TRP metabolites can modulate behavioral and patient-reported outcomes. We had few missing data, especially for urine KYNA and QUINA levels because of missing creatinine measurements. This could be avoided by assessing these outcomes in blood samples as well. Moreover, it will be important to analyze the influence of different exercise modalities on cytokines (such as IL-6, IFN-γ, and IL-10), and enzyme expression/activity (e.g., IDO1, KATs, and KMO) in different cell types and tissues (e.g., immune cells, muscle tissue, and tumor tissue) within the KYN pathway. A combined approach assessing acute and chronic effects of exercise-induced alterations of TRP metabolites on the frequencies and properties of specific immune cells (e.g., regulatory T-cells and NK-cells) could provide important mechanistic information on how exercise modulates tumor-relevant immune functions. In regards of the exercise program, endurance exercise or a combination of resistance and endurance exercise could be even more effective than resistance exercise alone. In fact, endurance exercise provokes different and more pronounced physiological adaptions, especially on the metabolic and immunological level. Investigating separate and additional effects of these exercise modalities should be essential next steps in this field of research. Since this study presents a secondary analysis, and therefore an explorative analysis of the BEST trial (35), we only had the option to investigate resistance exercise. Although BMI did not change over time in IG and CG, following studies should screen patients' nutrition status (e.g., intake of supplements) and provide more detailed information on body composition due to potential interactions with TRP metabolism.

In conclusion, the results of this investigation suggest that resistance exercise is able to sustainably reduce KYN levels in breast cancer patients undergoing radiotherapy. In light of the quest for inhibitors of the KYN pathway, resistance exercise may provide a non-pharmacological avenue through which cancer patients can actively reduce the flux through this tumor-promoting metabolic pathway.

## Data Availability Statement

The datasets generated for this study are available on request to the corresponding author.

## Ethics Statement

The studies involving human participants were reviewed and approved by the Ethics Committee of the University of Heidelberg (NCT01468766) and complied with the principles of the Declaration of Helsinki. The patients/participants provided their written informed consent to participate in this study.

## Author Contributions

KS, CU, JW, JD, and MS designed the umbrella study. CO, PZ, KS, KK, and MS designed this specific substudy. JW conducted the exercise intervention. CO, BB, and KK conducted and supported the lab work. JD recruited participants. PZ, CO, KS, MP, and MS performed statistical analysis and wrote the paper.

### Conflict of Interest

KK is the CEO of Neuroimmun GmbH, which produces and sells ELISA kits for the measurement of tryptophan, kynurenine, kynurenic acid, and quinolinic acid. The remaining authors declare that the research was conducted in the absence of any commercial or financial relationships that could be construed as a potential conflict of interest.
